# Peptide-Based Immunotherapeutics and Vaccines 2015

**DOI:** 10.1155/2015/349049

**Published:** 2015-12-20

**Authors:** Pedro Reche, Darren R. Flower, Masha Fridkis-Hareli, Yoshihiko Hoshino

**Affiliations:** ^1^Department of Microbiology-I/Immunology, Facultad de Medicina, Universidad Complutense, 28040 Madrid, Spain; ^2^School of Life and Health Sciences, Aston University, Birmingham B4 7ET, UK; ^3^ATR, LLC, Worcester, MA 01606, USA; ^4^Department of Mycobacteriology, National Institute of Infectious Diseases, Higashi-Murayama, Tokyo 189-0002, Japan

Vaccination produces profound and long lasting modifications in the adaptive immune system comprising T and B cells. Vaccines are curative not mere palliative remedies and thus vaccination is the most efficient method to prevent and to lesser extent treat infectious diseases, cancer, and allergy conditions. Currently, there is an increasing interest in developing vaccines based on synthetic peptides encompassing B and T cell epitopes that precisely trigger a protective immune response. Because they are synthetic, peptide vaccines are intrinsically safer than alternative vaccine formulations. Moreover, peptide-based vaccines will allow focusing solely on relevant epitopes, avoiding those that lead to nonprotective responses, immune evasion, or unwanted side effects, such as autoimmunity. However, developing a successful peptide-based vaccine requires addressing a number of significant difficulties, such as overcoming the low intrinsic immunogenicity of individual peptides. In this special issue on peptide-based vaccines, we have incorporated 9 original articles and two reviews that deal with and examine various aspects of peptide-based vaccine design.

The review by H. Kimura et al. offers new insights into the function of the immunoproteasome in immune and nonimmune cells. Cleavage of intracellular proteins by immunoproteasome is a key step on the MHC class I antigen presentation pathway and this review highlights the relevance of understanding immunoproteasome function for developing peptide-based vaccines and novel pharmacological treatments. The second review by Y.-F. Xiao et al. offers an outstanding analysis on peptide-based treatments for cancer. The authors divide peptide-based cancer treatments into three types, peptide-alone therapy, peptide vaccines, and peptide-conjugated nanomaterials, describing new advances in using peptides to treat lung, pancreatic, prostate, and gastric cancers. Moreover, the authors masterly collect evidence on how peptides represent ideal tumor immunotherapeutics as one can specifically target tumor cells with little toxicity and efficient immunoreaction.

The original articles incorporated in this issue consist of cutting-edge computational and experimental reports that are relevant for the design of peptide-based vaccines. Within the* in silico* manuscripts, we include a work by D. Kanduc et al. in which the authors compared the proteome of poliovirus with that of humans and found unique poliovirus peptide sequences that could be basis for developing a specific/universal vaccine, with no cross-reactions with human proteins. As an additional advantage, the authors argue that a peptide-based vaccine instead of current antipolio DNA vaccines would eliminate the rare postpoliomyelitis cases and other disabling symptoms that may appear following vaccination. Computer-assisted design of peptide-based vaccines often relies on more complex predictive models to select peptide fragments within antigens containing T and B cell epitopes. Generation of predictive models requires the assembling of categorized datasets for training the models. Dataset assembly is labor intense and time consuming. S. C. Pro et al. have masterly addressed the problem, devising a method to automate the generation of validated specific epitope sets from the IEDB database (http://www.iedb.org/). The availability of the disease-specific sequence data enables the use of predictive tools that reveal entire epitomes thus facilitating the development of epitope-based vaccines. However, in large and complex pathogens the potential T cell epitome can be so sizeable that it will challenge experimental validation. To address this problem M. Molero-Abraham et al. report a method and resource, EPIPOX, that allows downsizing the relevant T cell epitome for variola virus according to conservation criteria and antigen features such as expression and localization.

The experimental articles contained in the issue have a wide scope and include a phase II clinical trial by S. Yutani et al. The study is based on a personalized peptide vaccination with both a hepatitis C virus- (HCV-) derived peptide and peptides from tumor-associated antigens (TAA) for the treatment of HCV-positive advanced hepatocellular carcinoma patients. The authors show that the peptide-based vaccine (PPV) was safe and elicited HCV specific CTL responses as well as peptide-specific IgG1 responses to both the viral peptides and TAA-derived peptides supporting further clinical study of PPV. Identification of the antigens that are targeted by the immune system and characterizing the type of response are clearly a step forward towards designing a useful peptide-based vaccine. In this context, M. Niki et al. present an evaluation of humoral immunity to* Mycobacterium tuberculosis*- (TB-) specific antigens concluding that the induction of antigen-specific humoral immunity, especially for IgA response, is relevant for TB protection. Similarly, A. N. Kamali et al. show that* Plasmodium* antigens isolated by antibody affinity of sera from malaria-self-resistant ICR mice are capable of delaying infection when inoculated into BALB/c mice. Thereby, the authors conclude that immunoaffinity purified antigens using IgGs from protected individuals can be relevant for developing multiantigen blood-stage malaria vaccines. Vaccine development and optimization require finding appropriated animal models for testing. In this issue, I. Sominskaya et al. show that rabbits are suitable animal models to test the immunogenicity of core peptides from hepatitis C virus, promoting the use of rabbit models for preclinical trials of HCV vaccines.

Peptides exhibit little immunogenicity and thereby it is key to devise means of increasing their immunogenicity for vaccine design purposes. In this issue, A. Yano et al. show that combining amyloid beta (A*β*) peptides with toxoid (DT) enhanced the immunogenicity of the peptide on cynomolgus monkeys and guinea pigs that were first immunized with conventional diphtheria-tetanus. Moreover, the peptide vaccine induced anti-A*β* antibodies in cynomolgus monkeys and guinea pigs without chemical adjuvants, and excessive immune responses were not observed. Peptides can also have immunomodulatory properties as shown in the article by J.-P. Vernot et al., in which the authors show that it is possible to modulate p56Lck in T cells by a chimeric peptide comprising two functionally different motifs of Tip from* Herpesvirus saimiri.*

In conclusion, this special issue surveyed many aspects of peptide-based vaccines and we hope readers will find it both interesting and inspiring. It certainly has been a pleasure for us to select the work presented in this issue.

*Pedro Reche* *Pedro Reche* *Darren R. Flower* *Darren R. Flower* *Masha Fridkis-Hareli* *Masha Fridkis-Hareli* *Yoshihiko Hoshino* *Yoshihiko Hoshino*

